# Comparison of Objective and Perceived Access to Food Stores Associated with Intake Frequencies of Vegetables/Fruits and Meat/Fish among Community-Dwelling Older Japanese

**DOI:** 10.3390/ijerph16050772

**Published:** 2019-03-03

**Authors:** Miwa Yamaguchi, Katsuya Takahashi, Masamichi Hanazato, Norimichi Suzuki, Katsunori Kondo, Naoki Kondo

**Affiliations:** 1 Department of Epidemiology and Prevention, Center for Clinical Sciences, National Center for Global Health and Medicine, 1-21-1 Toyama, Shinjyuku, Tokyo 162-8655, Japan; 2 Policy Research Institute, Ministry of Agriculture, Forestry and Fisheries, 3-1-1, Kasumigaseki, Chiyoda-ku, Tokyo 100-0013, Japan; katsuyat@affrc.go.jp; 3 Center for Preventive Medical Sciences, Chiba University, Inage-ku Yayoi-cho 1-33, Chiba City 263-8522, Japan; hanazato@chiba-u.jp (M.H.); suzu-nori@chiba-u.jp (N.S.); kkondo@chiba-u.jp (K.K.); 4 Center for Well-Being and Society, Nihon Fukushi University, 5-22-35 Chiyoda, Naka-ku, Nagoya, Aichi 450-0003, Japan; 5 Department of Gerontological Evaluation, Center for Gerontology and Social Science, National Center for Geriatrics and Gerontology, 7-430 Morikoka-cho, Obu-shi, Aichi 474-8511, Japan; 6 Department of Health and Social Behavior, School of Public Health, The University of Tokyo, 7-3-1 Hongo, Bunkyo-ku, Tokyo 113-0033, Japan; naoki-kondo@umin.ac.jp; 7 Department of Health Education and Health Sociology, School of Public Health, The University of Tokyo, 7-3-1 Hongo, Bunkyo-ku, Tokyo 113-0033, Japan

**Keywords:** objective access, perceived access, vegetables/fruits intake, meat/fish intake

## Abstract

This cross-sectional study aimed to compare access to the nearest food stores with perceived access associated with intake frequencies of vegetables/fruits and meat/fish among older Japanese people. We used intake frequencies of vegetables/fruits and meat/fish from a self-administered questionnaire in the Japan Gerontological Evaluation Study among 83,384 adults aged over 65 years. We defined distance over 1 km as poor objective access in community level. We performed multilevel regression analysis to investigate the association of objective and perceived access with intake frequencies of vegetables/fruits and meat/fish, respectively. Participants who lived in poor objective access had a significantly higher intake frequency of vegetables/fruits than those who lived in good access. In contrast, residents with poor perceived access consumed lower frequent intake of vegetables/fruits (beta coefficient (standard error) 0.086 (0.021) for objective access; −0.093 (0.009) for perceived access). There was no significant association between objective access and intake frequency of meat/fish, but poor perceived access showed a significant association with lower intake frequency of meat/fish. There was inconsistency between objective and perceived measurement of access to food stores associated with dietary habits among older Japanese adults. Food access needs to be comprehensively assessed, while considering characteristics of measurements.

## 1. Introduction

Areas with poor food access, where it is relatively difficult to obtain healthy and affordable food, are referred to in Western countries as “food deserts” [1]. Previous reviews [1,2] have suggested that poor food access induces social disparities in diet-related health outcomes, such as obesity in relation to ethnic minorities and socioeconomically disadvantaged neighborhoods, especially in the United States and other developed Western countries. In Japan, it has been observed that approximately 35% of residents in a large city had poor access to fresh food as estimated using an objective information system (GIS) in line with a 2015 report [3]. Specifically, Japanese “food deserts” mainly affect older people residing in neighborhoods where smaller retail stores have closed because of the recent economic recession [4,5,6]. In Japanese urban or suburban areas, older adults often experience inconveniences when the stores were located more than 1 km away from their home [5]. The inconvenience was majorly induced by the physical burden of failing health and limited transportation [7]. Ikejima [3] reported approximately 35% of residents aged 65 years and older had poor access to fresh food in a large Japanese city. Therefore, the association between food access and dietary intake among older people has been given priority in public health research.

This study was designed to investigate the limited evidence on the association of food access with dietary intake, such as vegetables/fruits and meat/fish intake. First, Asian studies that used both GIS-based (objective) and perceived access and compared the association with dietary intake are scarce, although some western studies have been reported [8,9,10,11]. Objective access is limited in its ability to measure store utilization or residents’ true access to stores [9]. Therefore, it is important to investigate whether objective access is associated with individual dietary habits as well as perceived access. Second, empirical evidence of the association between food access and vegetables/fruits and/or meat/fish intake among community-based older adults had not yet been reported. Previous articles have targeted younger and middle-aged individuals [8,9,10,11,12,13,14], mixed race/ethnic populations [8,9,10,11,12,14,15], and people with low socioeconomic status [9,11,12,13,14]. Third, seven studies performed in western settings [8,9,10,11,12,14,15] did not show a consistent association between food access and vegetables/fruits intake. One study [10] showed that individuals were more likely to increase their servings per day of vegetables/fruits with increasing distance from a primary food store. However, two studies showed that individuals who lived more remotely with decreased access to food stores consumed significantly lower vegetables [12,15] and fruits [15] than those who lived in close proximity. While, four of the studies [8,9,11,14] reported no significant association between objective food access and vegetables/fruits intake. Fourth, most studies [1,2] used only vegetables and fruits as a measure of healthy dietary habits. It is important to investigate meat and fish intake in relation to food access because these foods are one of the protein-rich foods and associated with frailty prevention among older people [16]. Fifth, most previous studies have investigated the association between food access and dietary intake in only urban/suburban areas [8,9,10,11,12,13] or rural areas [15], except for a study by Pearce et al. [14] that adjusted for urban and rural areas, as a higher number of food markets with fresh vegetables/fruits and meat/fish are generally located in urban areas rather than in rural areas [17]. Finally, although some Japanese studies have reported an association between objective and perceived access and health outcomes [18,19,20], more evidence is required. A recent study showed that lower availability of healthy food stores measured subjectively, but not objectively, was associated with mortality [20]. Regarding nutritional status, objective [18] and subjective [19] food access showed a culture-specific association with being obese or underweight among Japanese older adults compared to western settings [1,2]. As these two studies [18,19] investigated a limited region, studies involving a larger-scale region are needed.

Our aim was to compare objective and perceived access associated with intake frequencies of vegetables/fruits and meat/fish among Japanese community-dwelling older people. As an additional investigation, we repeated the analyses stratifying by urban/suburban and rural areas. Furthermore, we analyzed the association of objective and perceived access with the prevalence of underweight and overweight individuals. In addition, to confirm the availability of healthy food, we investigated the association of intake frequencies of vegetables/fruits and meat/fish with body mass index (BMI) which is one of the measures of nutritional status.

## 2. Materials and Methods

### 2.1. Study Participants

Respondents were identified from the Japan Gerontological Evaluation Study (JAGES), a prospective cohort study investigating the influence of the social determinants of health outcomes among older individuals aged 65 years or older in Japan [21,22]. For this study, we used cross-sectional data from the 2010–2011 survey (response rate: 66.3%). Participants who did not receive long-term care and resided in 31 municipalities in 12 of 47 prefectures in Japan were included. Of 102,869 potential participants, we excluded those with missing information on school districts (*n* = 4099), and those who lived in school districts with fewer than 50 residents (*n* = 5134) [23]. We used school districts as the smallest area unit available in the JAGES [22]. Historically, school districts were used to represent the former unit of “villages” before repeated municipality mergers took place in the last few decades in Japan [24].

**Figure 1:**
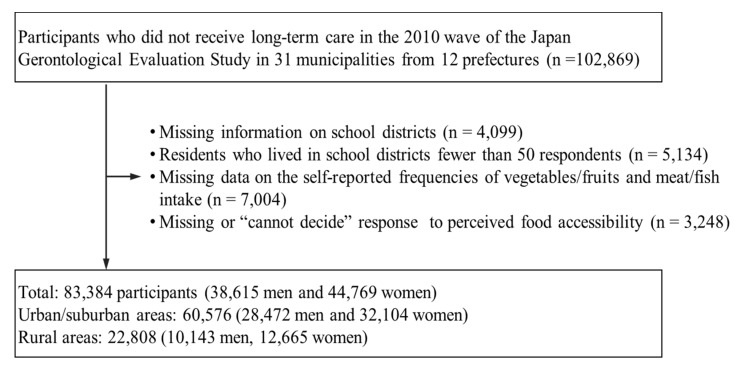
Flow diagram of the study participants.

After excluding individuals with missing data on the self-reported frequencies of vegetables/fruits and meat/fish intake (*n* = 7004) and missing or “I don’t know” response to perceived food accessibility (*n* = 3248) in the questionnaire, a total of 83,384 participants (38,615 men and 44,769 women) who resided in 426 school districts across 29 municipalities were included in the subsequent analyses (Figure 1). Of those, the number of individuals in urban/suburban areas and rural areas were 60,576 (28,472 men and 32,104 women) and 22,808 (10,143 men, 12,665 women), respectively. This study was conducted in accordance with the Declaration of Helsinki. The JAGES protocol and informed consent procedures were approved by the Ethics Committee for Research of Human Subjects at Nihon Fukushi University (no. 10-05 and no. 13-14) and the Ethics Committee for Medical Research at the University of Tokyo (no. 10555).

### 2.2. Objective Access

Food access was estimated using a GIS map created by the Policy Research Institute, Ministry of Agriculture, Forestry and Fisheries of Japan [5]. First, we created half-grid square data of the population from the 2010 national census obtained from the Statistical Information Institute for Consulting and Analysis [25] and the number of food stores, including large-scale department stores, giant or small supermarkets, and specialty shops (those selling vegetables, fruits, meat, and fish). Convenience stores were excluded based on wholesale or retail sales data from the Current Survey of Commerce, in 2007 [26]. Second, we calculated the probability of the population accessing the nearest food store at a specific distance from a residence, assuming that the population and stores were distributed uniformly within the half-grid square [5]. The probability of the population accessing stores was estimated every 100 m for a 0–1.9-km radius centered on the point of residence, every 1 km for a 2.0–19.9-km radius, and every 10 km for a 20–70-km radius. Third, we calculated the probabilities of the population accessing the nearest food store between each radii rage centered on the point of residence as described above. Next, we estimated the weighted average of the distance to the nearest food store within the half-grid square. Examples of the processed model include the following:

the probability of the population accessing the nearest food stores located within a 50-m distance (i.e., midpoint of the radius between 0 and 100 m centered on the point of residence)
  over 0 m − over 100 m = 100% − 97.6% = 2.4%;
the probability of the population accessing the nearest food stores located within 2.5 km
  over 2 km − over 3 km = 12.0% − 9.4% = 2.6%;
the probability of the population accessing the nearest food stores located within 65 km
  over 60 km − over 70 km = 0.1% − 0.0% = 0.1%;
and the weighted average of the distance to the nearest stores within the half-grid square
  = (50 m × 2.4% + 2.5 km × 2.6%+ 65 km × 0.1%)/(50 m + 2.5 km + 65 km).

**Figure 2:**
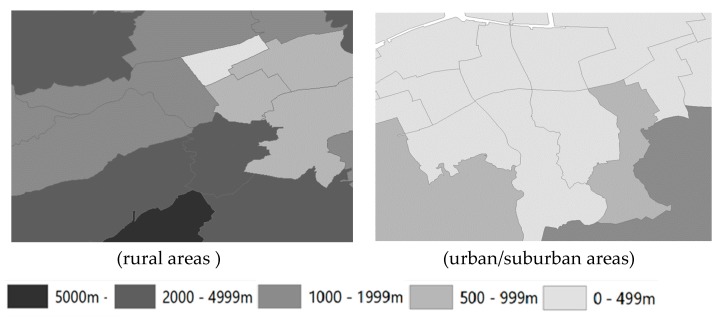
Estimated distance to the nearest food store (food access (m)) by school district. Values on the maps represent food access in each school district in rural areas (**left**) and urban/suburban areas (**right**). As indicated, the school district was colored from light-gray to dark-gray as the distances to stores increased.

Finally, the weighted average of the distance to the nearest food stores within the half-grid square was aggregated and averaged within the school districts as an indicator of food access at the community level (Figure 2). Areas with a population density greater than 4000 population/km^2^ were defined as urban/suburban areas, and those with a lower population density were defined as rural areas according to the Statistics Bureau [27]. The mean population densities of the school districts established by the JAGES [28,29], calculated in 486 school districts in urban/suburban areas and in 95 school districts in rural areas, were 7442 and 499 population/km^2^, respectively. This study used binominal food access to define groups of a distance of less than and over 1 km as good and poor food access, respectively [19].

### 2.3. Perceived Access

The measurement of perceived access in the JAGES study has been used previously [19,20]. The perceived availability of food was assessed using the question “How many stores or facilities selling fresh fruits and vegetables are located within 1 kilometer of your home?”. The following responses were given on a four-point Likert scale: “Many”, “Some”, “Few”, “None”, or “I don’t know”. Subjects who answered “Many” or “Some” were categorized as having high access, and respondents who answered “Few”, or “None”, were categorized as having low access. For a more a precise assessment, we excluded participants who responded “I don’t know” in this study.

### 2.4. Intake Frequencies of Vegetables/Fruits and Meat/Fish

We used responses about average intake of vegetables/fruits and meat/fish over a one-month period among participants as previous study used [19]. The intake frequencies were stratified into the following categories: every day and over twice/day, every day and once/day, 4–6 times/week, 2–3 times/week, once-a-week, less than once-a-week, and almost never. In this analysis, we assigned scores of 2, 1, 0.7, 0.4, 0.1, 0.05, and 0 (times/day), respectively, to each of the categories.

### 2.5. Covariates

The number of convenience stores in each school district was used as a covariate, as data on current food access were not included due to technical issues. Further, the degree of land slope in the neighborhood (continuous value) and car use by individuals, family members, or friends driving (yes or no) were significant covariates in terms of food access [18,30]. The average land slope at a community level was calculated by using the national dataset from the Ministry of Land, Infrastructure, Transport and Tourism in Japan, based on the Digital Map 50 m Grid (Elevation) from the GIS [31]. To consider the area difference, we used urban/suburban and rural areas as a covariate. We further considered the following covariates in this study: age (65–69, 70–74, 75–79, or ≥80 years), sex (men or women), family structure (living alone, with a spouse, or with others), BMI (<18.5, 18.5–24.9, or ≥25 kg/m^2^), marital status (married, divorced, widowed, or never married), activities of daily living (ADLs) (<5 or 5 units), the number of remaining teeth (≥20 or <19), presence of comorbidities (yes or no), smoking status (current, past, or never), household income (<2.00, 2.00–3.99, or ≥4.00 million yen), and years of schooling (<9, 10–12, or ≥13 years). The unknown variables were treated as categorical data to examine any associations between food access and the frequency of vegetables/fruits and meat/fish intake. BMI was calculated as the body weight in kilograms divided by the square of the body height in meters. ADLs were assessed by five items: use of public transportation, shopping for daily necessities, preparing meals, paying bills, and managing bank deposits [32]. The annual normalized household income was determined from the total household income divided by the square root of the number of household members as an equivalent household income. In terms of comorbidities, respondents were asked if they were currently under medical treatment for any of the following conditions (all of which may confound vegetable/fruit and meat/fish intake): cancer, heart disease, stroke, hypertension, diabetes mellitus, obesity, hyperlipidemia, osteoporosis, gastrointestinal disease, mental disorders, or dysphagia [33].

### 2.6. Statistical Analyses

By using food access (poor vs. good) of objective measurement on a community level and perceived measurement on an individual level as binomial explanatory variables, we performed a multilevel Tobit model, adjusting for all covariates, to estimate the standardized beta (*β*) coefficient and standard error (SE) for the intake frequency of vegetables/fruits and meat/fish. Based on the random-effects variance, we calculated the intraclass correlation (ICC) to examine the proportion of the variance in dietary habits (i.e., intake frequencies of vegetables/fruits and meat/fish) that occurs at the neighborhood (i.e., school district) level [34]. An ICC equal to 1 would inform us that all the people in a neighborhood have identical dietary habits, and an ICC equal to 0 would indicate that the people do not share any neighborhood related those at a common level. As additional analyses, we repeated the analyses stratifying by urban/suburban and rural areas. Furthermore, a multilevel logistic regression model was performed to investigate the associations of objective and perceived access with the prevalence of underweight (BMI < 18.5 kg/m^2^) and overweight (BMI ≥ 25 kg/m^2^) among 80,012 residents without missing variable of BMI. We also investigated the association of intake frequencies of vegetables/fruits and meat/fish with BMI by using a multilevel regression model adjusted for age and sex. Statistical significance was set as a two-sided *p*-value of 0.05. All statistical analyses were conducted by using Stata (ver. 15.0; StataCorp, College Station, TX, USA).

## 3. Results

The proportion of individuals with poor food access was 36.4% (30,383 individuals) by objective measurement and 25.3% (21,105 individuals) by perceived measurement (Table 1). The average age was approximately 74 years old in all participants. Most of residents in poor objective and perceived access lived with others, and were likely to be married, BMI 18.5–24.9 kg/m^2^, <5 units of activity daily living, <20 of remaining teeth, having comorbidity, past or never smoking, <2.00 million yen/year of household income, ≤9 years of schooling, and using a car. The 36.6% residents in poor objective access and 61.8% residents in poor perceived access lived in urban/suburban areas. In community level, there were approximately 2–3 convenience stores in poor objective and perceived access. A steeper land slope in poor access was observed in objective and perceived access and was clearly observed in objective poor access.

The associations of poor food access (vs. good) with the intake frequency of vegetables/fruits and meat/fish are shown in Table 2. Individuals who lived in poor objective access had significantly higher intake frequency of vegetables/fruits than those who lived in areas with good objective access (*β* = 0.086 (SE) 0.021). In contrast, individuals with poor perceived access had significantly lower intake frequency of vegetables/fruits than those living in good ones (*β* = −0.093 (SE) 0.009). There was no significant association between poor objective access and the intake frequency of meat/fish, but a significant inverse association was observed for perceived access (*β* = −0.029 (SE) 0.004).

When we performed stratification by urban/suburban and rural areas, associations of objective and perceived access with the intake frequency of vegetables/fruits and meat/fish did not change significantly. The association of objective and perceived access with the intake frequency of vegetables/fruits in urban/suburban areas was greater than those in rural areas (Tables S1–S4). As shown in Table S5, poor objective access compared to good food access was significantly associated with a lower prevalence of underweight individuals. However, poor perceived access was weakly associated with higher prevalence of underweight individuals compared to good food access. No significant association was observed between food access and the prevalence of obesity for both objective and perceived access. Regarding the association between intake frequencies of vegetables/fruits, meat/fish, and body mass index, a significant association (*p*-value < 0.001) was observed (Table S6).

## 4. Discussion

The present study found that the intake frequency of vegetables/fruits in poor objective access areas was significantly higher than that in good access areas among Japanese older people, on the contrary to the inverse association using perceived access. There was no significant association between objective access and intake frequency of meat/fish; however, poor perceived access was associated with lower intake frequency of meat/fish. To our knowledge, this is the first study to compare the measurement between objective and perceived access in relation to intake frequencies of vegetables/fruits and meat/fish among Japanese older people in a large-scale population-based study.

Our finding is in line with another study [10] that evaluated urban senior citizens in the United States, that suggested an increase, which was not statistically significant, in servings per day of vegetables/fruits for every 10th of a mile in distance to a primary food store. However, two studies among rural seniors [15] and urban residents aged over 16 years [12] showed that individuals who lived in poor objective access areas consumed significantly lower vegetables [12,15] and fruits [15] than those who lived in good access areas. Several studies have reported no significant association between objective access and vegetables/fruits intake among low-income and/or urban residents including younger people [8,9,11,14]. The inconsistencies in the results of this study and those of previous western studies [8,9,11,12,14,15] could be partly explained by culture-specific food environments, and populations with comparably younger ages, low income, and minority race/ethnicity.

This study found that, contrary to perceived access, those with poor objective access had significantly higher intake frequency of vegetables/fruits than those with good access. Objective access might not correctly reflect actual individual food purchasing behaviors better than perceived ones [9,11]. Especially in urban/suburban areas, residents with poor perceived access did not necessarily live in areas with poor objective access in this study. Urban residents are more likely to travel beyond their nearest supermarkets due to their demands, such as healthy foods [10] and low-cost foods [8]. Of those with poor objective access, 65% residents had good perceived access in this study. The residents with good perceived access in the poor objective access group might lead to higher intake frequency of vegetables/fruits. Some latent components that could not be incorporated with the objective access might result in the inconsistency of results between objective and perceived access in this study. Among latent components that could not be incorporated with the objective access, we suggest the possible components from Japanese specific food environment as follows. First, objective access might not appropriately capture that older adults may obtain vegetables/fruits at small local markets that were not identified by national data of food markets against perceived access. Second, there is a possibility that land uses for agriculture or fishing confounded the association between objective access and dietary intake. With the presence of land use for agriculture or fishing, residents might find it easier to obtain vegetables/fruits and fish through small farmer’s markets and/or food exchanges [35,36] than those who lived in other areas. Third, online shopping, home delivery service, a food vendor vehicle service, and small retail shop provided by the local government, nongovernmental organization, social organizations, or large retail companies (i.e., convenient stores) increased in number to support older residents who lived in areas lacking food stores since around 2010 [7]. These services may attenuate the inconvenience of food access due to the distance to the food stores.

As previous studies did not focus on meat/fish intake in relation to objective and perceived access, the present findings could not be compared with other studies. The associations between objective and perceived access and intake frequency of meat/fish in this study indicated a similar trend but a weaker association compared to those of vegetables/fruits. We suggest that the association of food access with the intake frequency of meat/fish could be explained with similar reasons as those mentioned for vegetables/fruits above.

This study showed poor objective access was significantly associated with lower prevalence of underweight. In contrast to our result, Hanibuchi et al. [18] found that the number of supermarkets on an individual level was negatively associated with being underweight, although this was not statistically significant. Furthermore, this study showed poor objective access was weakly associated with a higher prevalence of overweight compared to those with good access. However, the previous study [18] showed that there was a significant negative association between the distance to the nearest supermarket and overweight/obesity. The association between perceived poor food access and higher prevalence of underweight individuals in this study was consistent with the study by Nakamura et al. [19]; however, there was no significant association due to the limited regions. Although further prospective studies are warranted, it may be necessary to support older residents with poor perceived access to prevent underweight.

Our study has the following strengths. First, we employed an accurate food access map from national census data compiled by the Policy Research Institute, Ministry of Agriculture, Forestry and Fisheries of Japan [3]. Second, we adjusted for the number of convenience stores and the grade of the land slope in the school districts as confounding factors in the association between food access and dietary intake. Third, our results have generalizability, since the data was collected from a large-scale investigation conducted in both urban/suburban and rural areas.

Nevertheless, this study has some limitations. First, national data for assessing objective access might not be able to identify small food markets and non-market-based food access (e.g., exchanging with neighbors and making home gardens) [35,36]. This limitation may lead to the underestimation of objective access. Second, the perceived measurement we used was not validated. However, we assessed perceived access to be comparably accurate according to the hillier environments where residents with poor perceived access lived. Older adults with poor perceived access were likely to have a lower level health status than those who reported good access in this study. This characteristic was consistent to that reported in a previous study [7] which reported that older people who live in food desert areas suffer from the physical burden of failing health when trying to access groceries. Therefore, the perceived access can accurately describe the accessibility to food stores. Third, we measured dietary habits using only data regarding vegetables/fruits and meat/fish intake. We did not investigate the validity of self-reported intake frequencies of vegetables/fruits and meat/fish using a dietary record. However, population-based studies [19,33] have used simple measures to assess intake frequencies of vegetables/fruits and meat/fish, representing a limitation of this field of research. In addition, we confirmed that intake frequencies of vegetables/fruits and meat/fish were available for assessing healthy food by confirming a significant association with BMI. Nevertheless, Japanese people usually consume a variety of foods [37], which are purchased at food stores. Therefore, the association between food access and dietary habits could have been identified more clearly if we measured the dietary diversity among older adults [38]. Fourth, this study had no information as to whether older adults reported intake frequencies of vegetables/fruits and meat/fish purchased through take-out meals or through informal networks. As this study did not measure the amounts of vegetables/fruits and meat/fish in cooked meals, the energy intake from vegetables/fruits and meat/fish may be under- or over-estimated. Finally, due to the cross-sectional design, causality could not be evaluated.

## 5. Conclusions

We found that there existed inconsistency between objective and perceived measurement of access to food stores associated with intake frequencies of vegetables/fruits and meat/fish among older Japanese adults. Food access should be comprehensively assessed, taking into account the characteristics of measurement of food access. In the future, we should perform prospective studies to investigate the association between food access and dietary habits affected by several factors including affordability, accommodation, and acceptability in addition to GIS-based measures [39]. Using these assessments, it is important to decide what dimension of food access we should support with priority for older adults.

## Figures and Tables

**Table 1:** Characteristics of 83,384 participants by objective and perceived access.

	Total ^a^ *n* = 83,384	Poor Objective Access *n* = 30,383	Poor Perceived Access *n* = 21,105
Objective access: poor, *n* (%)	30,383	-	10,696 (50.7)
Perceived access: poor, *n* (%)	21,105	10,696 (35.2)	-
Age (years), mean (SD)	73.9 (6.2)	74.5 (6.4)	74.2 (6.4)
Men, *n* (%)	38,615	13,604 (44.8)	8954 (42.3)
Family structure, *n* (%)			
Alone	9896	3513 (11.6)	2849 (13.5)
With their spouse	31,151	10,876 (35.8)	7463 (35.4)
With others	41,139	15,485 (51.0)	10,456 (49.5)
Unknown	1198	509 (1.7)	337 (1.6)
Marital status, *n* (%)			
Married	59,313	21,227 (69.9)	14,199 (67.3)
Divorced or widowed	20,740	7926 (26.1)	6005 (28.5)
Never married or others	2036	616 (2.0)	544 (2.6)
Unknown	1295	614 (2.0)	357 (1.7)
Body mass index (kg/m^2^), *n* (%)			
<18.5	5759	1987 (6.5)	1556 (7.4)
18.5–24.9	56,671	20,316 (66.9)	14,138 (67.0)
≥25	15,917	6572 (21.6)	4396 (20.8)
Unknown	1665	1508 (5.0)	1015 (4.8)
Activity daily living (units), *n* (%)			
<5	16,926	6430 (21.2)	4944 (23.4)
≥5	64,453	23,071 (75.9)	15,577 (73.8)
Unknown	2005	882 (2.9)	584 (2.8)
Remaining teeth (number), *n* (%)			
<20	54,238	21,697 (71.4)	14,407 (68.3)
≥20	27,535	8040 (26.5)	6267 (29.7)
Unknown	1611	646 (2.1)	431 (2.0)
Comorbidity, *n* (%)			
No	11,491	4107 (13.5)	3052 (14.5)
Yes	52,049	19,206 (63.2)	13,409 (63.5)
Unknown	19,844	7070 (23.3)	4644 (22.0)
Smoking status			
Current	8735	2977 (9.8)	2125 (10.1)
Past or never	69,660	25,041 (82.4)	17,496 (82.9)
Unknown	4989	2365 (7.8)	1484 (7.0)
Household income (million yen/year), *n* (%)			
<2.00	34,481	13,887 (45.7)	9266 (43.9)
2.00–3.99	26,667	8442 (27.8)	6136 (29.1)
≥4.00	7649	2048 (6.7)	1669 (7.9)
Unknown	14,587	6006 (19.8)	4034 (19.1)
Years of schooling (years), *n* (%)			
≤9	38,634	15,225 (50.1)	10,336 (49.0)
10–12	28,557	10,058 (33.1)	7012 (33.2)
≥13	14,267	4328 (14.2)	3201 (15.2)
Unknown	1926	772 (2.5)	556 (2.6)
Car use, *n* (%)			
Yes	61,925	26,125 (86.0)	16,270 (77.1)
No	12,550	3729 (12.3)	3099 (14.7)
Unknown	8909	529 (1.7)	1736 (8.2)
Community level			
Urban/suburban area, *n* (%)	60,576	11,107 (36.6)	13,044 (61.8)
Convenience stores (number), mean (SD)	3.6 (3.1)	2.3 (2.1)	3.0 (3.0)
Land slope (degree), mean (SD)	4.9 (5.9)	9.4 (7.0)	6.7 (7.2)
Vegetables/fruits intake (times/day), mean (SD)	1.4 (0.6)	1.4 (0.6)	1.3 (0.6)
Meat/fish intake (times/day), mean (SD)	0.8 (0.5)	0.8 (0.5)	0.7 (0.5)

SD = standard deviation; ^a^ Numbers or mean (SD) were indicated in Total.

**Table 2:** Intake frequencies of vegetables/fruits and meat/fish according to objective and perceived access.

	Objective Access		Perceived Access	
	*β* (SE)	*p*-Value	*β* (SE)	*p*-Value
**Vegetables/fruits**				
Poor access (vs. good access)	0.086 (0.021)	<0.001	−0.093 (0.009)	<0.001
Age	0.018 (0.001)	<0.001	0.018 (0.001)	<0.001
Men (vs. women)	−0.376 (0.009)	<0.001	−0.379 (0.009)	<0.001
Living alone (vs. with others)	0.041 (0.014)	0.004	0.042 (0.014)	0.003
Never married or others (vs. married)	−0.184 (0.026)	<0.001	−0.182 (0.026)	<0.001
BMI (vs. 18.5–24.9 kg/m^2^)				
<18.5	−0.010 (0.015)	0.494	−0.010 (0.015)	0.528
≥25	−0.096 (0.009)	<0.001	−0.096 (0.009)	<0.001
Activity daily living, <5 units (vs. ≥5)	−0.137 (0.010)	<0.001	−0.132 (0.010)	<0.001
Remaining teeth, <20 tooth (vs. ≥20)	−0.217 (0.009)	<0.001	−0.215 (0.009)	<0.001
Comorbidity, yes (vs. no)	−0.028 (0.011)	0.015	−0.028 (0.011)	0.013
Current smoking (vs. never)	−0.243 (0.012)	<0.001	−0.243 (0.012)	<0.001
Household income, <2.00 million yen/year (vs. 2.00–3.99)	−0.173 (0.009)	<0.001	−0.171 (0.009)	<0.001
Years of schooling, <9 years (vs. 10–12)	−0.133 (0.009)	<0.001	−0.131 (0.009)	<0.001
No car-use (vs. use)	−0.133 (0.011)	<0.001	−0.135 (0.011)	<0.001
Urban/suburban area (vs. rural area)	0.004 (0.023)	0.850	−0.050 (0.021)	0.017
Convenience store	−0.002 (0.002)	0.276	−0.003 (0.002)	0.145
Land slope	−0.001 (0.001)	0.346	0.001 (0.001)	0.319
	Var RE (SE)	ICC (SE)	Var RE (SE)	ICC (SE)
	0.008 (0.001)	0.008 (0.001)	0.010 (0.001)	0.010 (0.001)
**Meat/fish**				
Poor access (vs. good access)	0.021 (0.014)	0.130	−0.029 (0.004)	<0.001
Age	0.005 (0.0003)	<0.001	0.005 (0.0003)	<0.001
Men (vs. women)	−0.075 (0.004)	<0.001	−0.076 (0.004)	<0.001
Living alone (vs. with others)	−0.025 (0.007)	<0.001	−0.025 (0.007)	<0.001
Never married or others (vs. married)	−0.057 (0.012)	<0.001	−0.057 (0.012)	<0.001
BMI (vs. 18.5–24.9 kg/m^2^)				
<18.5	0.005 (0.007)	0.486	0.005 (0.007)	0.459
≥25	−0.019 (0.004)	<0.001	−0.019 (0.004)	<0.001
Activity daily living, <5 units (vs. ≥5)	−0.042 (0.005)	<0.001	−0.040 (0.005)	<0.001
Remaining teeth, <20 tooth (vs. ≥20)	−0.082 (0.004)	<0.001	−0.082 (0.004)	<0.001
Comorbidity, yes (vs. no)	−0.008 (0.005)	0.135	−0.008 (0.005)	0.126
Current smoking (vs. never)	−0.014 (0.006)	0.017	−0.014 (0.006)	0.018
Household income, <2.00 million yen/year (vs. 2.00–3.99)	−0.080 (0.004)	<0.001	−0.079 (0.004)	<0.001
Years of schooling, <9 years (vs. 10–12)	−0.081 (0.004)	<0.001	−0.081 (0.004)	<0.001
No car-use (vs. use)	−0.020 (0.005)	<0.001	−0.021 (0.005)	<0.001
Urban/suburban area (vs. rural area)	0.037 (0.015)	0.014	0.023 (0.013)	0.080
Convenience store	−0.0004 (0.001)	0.704	−0.001 (0.001)	0.596
Land slope	0.001 (0.001)	0.102	0.002 (0.001)	0.011
	Var RE (SE)	ICC (SE)	Var RE (SE)	ICC (SE)
	0.005 (0.0005)	0.019 (0.002)	0.005 (0.0005)	0.019 (0.002)

*β* = beta coefficients; SE = standard error; Var RE = random effect variance in 426 school districts; ICC = intercorrelation between school districts; Associations were assessed using a multilevel Tobit model among 83,384 residents: Var RE (SE) and ICC (SE) in the null model was 0.016 (0.002) and 0.014 (0.002) for vegetables/fruits and 0.006 (0.001) and 0.025 (0.002) for meat/fish, respectively.

## Supplementary Materials

The supplementary materials can be found at https://www.mdpi.com/1660-4601/16/5/772/s1. Table S1: Characteristics of participants by objective and perceived access in urban/suburban areas, Table S2: Characteristics of participants by objective and perceived access in rural areas, Table S3: Intake frequencies of vegetables/fruits and meat/fish according to objective and perceived access in suburban/urban areas, Table S4: Intake frequencies of vegetables/fruits and meat/fish according to objective and perceived access in rural areas, Table S5: Evaluation of the association of food access with the prevalence of underweight and overweight individuals compared to those with a normal weight, Table S6: Evaluation of the association of intake frequencies of vegetables/fruits and meat/fish with body mass index.
