# Supplementary material for: Comparison of Objective and Perceived Access to Food Stores Associated with Intake Frequencies of Vegetables/Fruits and Meat/Fish among Community-Dwelling Older Japanese
Source: Int J Environ Res Public Health. 2019 Mar 3;16(5):772. doi: 10.3390/ijerph16050772 (PMC6427395; doi:10.3390/ijerph16050772)
Supplement: Supplementary file 1 [file ijerph-16-00772-s001.pdf]

Additional file 1

Table S1 Characteristics of participants by objective and perceived access in urban/suburban areas

	Total[a] n = 60576	Poor objective access n = 11107	Poor perceived access n = 13044
Objective access: poor, n (%)	11107	-	3385 (26.0)
Perceived access: poor, n (%)	13044	3385 (30.5)	-
Age (years), mean (SD)	73.6 (6.0)	73.7 (6.2)	73.7 (6.3)
Men, n (%)	28472	5093 (45.9)	5614 (43.0)
Family structure, n (%)			
Alone	7198	1136 (10.3)	1749 (13.4)
With their spouse	23166	4068 (36.6)	4632 (35.5)
With others	29436	5762 (51.9)	6480 (49.7)
Unknown	776	141 (1.3)	183 (1.4)
Marital status, n (%)			
Married	43476	7919 (71.3)	8843 (67.8)
Divorced or widowed	14743	2803 (26.6)	3644 (27.9)
Never married or others	1570	208 (1.9)	377 (2.9)
Unknown	787	177 (1.6)	180 (1.4)
Body mass index (kg/m^2), n (%)			
< 18.5	4221	692 (6.2)	991 (7.6)
18.5–24.9	41537	7534 (67.8)	8800 (67.5)
≥ 25	12690	2420 (21.8)	2717 (20.8)
Unknown	2128	461 (4.2)	536 (4.1)
Activity daily living (units), n (%)			
< 5	11888	2244 (20.2)	2985 (22.9)
≥ 5	47417	8614 (77.6)	9754 (74.8)
Unknown	1271	249 (2.2)	305 (2.3)
Remaining teeth (number), n (%)			
< 20	37330	7184 (64.7)	8313 (63.7)
≥ 20	22186	3748 (33.7)	4499 (34.5)
Unknown	1060	175 (1.6)	232 (1.8)
Comorbidity, n (%)			
No	8303	1424 (12.8)	1843 (14.1)
Yes	37707	7050 (63.5)	8326 (63.8)
Unknown	14566	2633 (23.7)	2875 (22.0)
Smoking status			
Current	6553	1169 (10.5)	1426 (10.9)
Past or never	50996	9243 (83.2)	10890 (83.5)
Unknown	3027	695 (6.3)	728 (5.6)
Household income (million yen/year), n (%)			
< 2.00	23772	4613 (41.5)	5323 (40.8)
2.00–3.99	20885	3700 (33.3)	4234 (32.5)
≥ 4.00	6156	874 (7.9)	1240 (9.5)
Unknown	9763	1920 (17.3)	2247 (17.0)
Years of schooling (years), n (%)			
≤ 9	26225	4900 (44.1)	5950 (45.6)
10–12	21632	4092 (36.8)	4573 (35.1)
≥ 13	11441	1901 (17.1)	2220 (17.2)
Unknown	1278	214 (1.9)	301 (2.3)
Car use, n (%)			
Yes	42168	9408 (84.7)	9358 (71.7)
No	9868	1495 (13.5)	2105 (16.1)
Unknown	8540	204 (1.8)	1581 (12.1)
Community level			
Convenience stores (number), mean (SD)	3.7 (2.0)	3.7 (2.0)	4.2 (3.1)
Land slope (degree), mean (SD)	2.8 (3.8)	5.7 (5.6)	3.1 (4.0)
Vegetables/fruits intake (times/day), mean (SD)	1.4 (0.6)	1.4 (0.6)	1.3 (0.6)
Meat/fish intake (times/day), mean (SD)	0.8 (0.5)	0.8 (0.5)	0.7 (0.5)

SD = standard deviation; [a] Numbers or mean (SD) were indicated in Total.

Additional file 1

Table S2 Characteristics of participants by objective and perceived access in rural areas

	**Total[a] n = 22808**	**Poor objective access n = 19276**	**Poor perceived access n = 8061**
Objective access: poor, n (%)	19276	-	7311 (90.7)
Perceived access: poor, n (%)	8061	7311 (37.9)	-
Age (years), mean (SD)	74.8 (6.5)	75.0 (6.5)	75.1 (6.5)
Men, n (%)	10143	8511 (44.2)	3340 (41.4)
Family structure, n (%)			
Alone	2698	2377 (12.3)	1100 (13.7)
With their spouse	7985	6808 (35.3)	2831 (35.1)
With others	11703	9723 (50.4)	3976 (49.3)
Unknown	422	368 (1.9)	154 (1.9)
Marital status, n (%)			
Married	15837	13308 (69.0)	5356 (66.4)
Divorced or widowed	5997	5123 (25.8)	2361 (29.3)
Never married or others	466	408 (2.1)	167 (2.1)
Unknown	508	437 (2.3)	177 (2.2)
Body mass index (kg/m^2), n (%)			
< 18.5	1538	1295 (6.7)	565 (7.0)
18.5–24.9	15134	12782 (66.3)	5338 (66.2)
≥ 25	4892	4152 (21.5)	1679 (20.8)
Unknown	1244	1047 (5.4)	479 (5.9)
Activity daily living (units), n (%)			
< 5	5038	4186 (21.7)	1959 (24.3)
≥ 5	17036	14457 (75.0)	5823 (72.2)
Unknown	734	633 (3.3)	279 (3.5)
Remaining teeth (number), n (%)			
< 20	16908	14513 (75.3)	6094 (75.6)
≥ 20	5349	4292 (22.3)	1768 (21.9)
Unknown	551	471 (2.4)	199 (2.5)
Comorbidity, n (%)			
No	3188	2683 (13.9)	1209 (15.0)
Yes	14342	12156 (63.1)	5083 (63.1)
Unknown	5278	4437 (23.0)	1769 (22.0)
Smoking status			
Current	2182	1808 (9.4)	699 (8.7)
Past or never	18664	15798 (82.0)	6606 (82.0)
Unknown	1962	1670 (8.7)	756 (9.4)
Household income (million yen/year), n (%)			
< 2.00	10709	9274 (48.1)	3943 (48.9)
2.00–3.99	5782	4742 (24.6)	1902 (23.6)
≥ 4.00	1493	1174 (6.1)	429 (5.3)
Unknown	4824	4086 (21.2)	1787 (22.2)
Years of schooling (years), n (%)			
≤ 9	12409	10325 (53.6)	4386 (54.4)
10–12	6925	5966 (31.0)	2439 (30.3)
≥ 13	2826	2427 (12.6)	981 (12.2)
Unknown	648	558 (2.9)	255 (3.2)
Car use, n (%)			
Yes	19757	16717 (86.7)	6912 (85.8)
No	2682	2234 (11.6)	994 (12.3)
Unknown	369	325 (1.7)	155 (1.9)
Community level			
Convenience stores (number), mean (SD)	1.6 (1.7)	1.5 (1.8)	1.0 (1.3)
Land slope (degree), mean (SD)	10.3 (6.9)	11.5 (6.8)	12.5 (7.4)
Vegetables/fruits intake (times/day), mean (SD)	1.4 (0.6)	1.4 (0.6)	1.4 (0.6)
Meat/fish intake (times/day), mean (SD)	0.7 (0.5)	0.8 (0.5)	0.7 (0.5)

SD = standard deviation; [a]Numbers or mean (SD) were indicated in Total.

Additional file 1

Table S3 Suburban/urban area intake frequencies of vegetables/fruits and meat/fish according to objective and perceived access

	**Objective access**		**Perceived access**	
	β **(SE)**	***p*-Value**	***β*** **(SE)**	***p*-Value**
Vegetables/fruits				
Poor access (vs. good access)	0.102 (0.021)	<0.001	-0.112 (0.011)	<0.001
Age	0.018 (0.001)	<0.001	0.018 (0.001)	<0.001
Men (vs. women)	-0.403 (0.010)	<0.001	-0.407 (0.010)	<0.001
Living alone (vs. with others)	0.059 (0.017)	<0.001	0.060 (0.017)	<0.001
Never married or others (vs. married)	-0.186 (0.030)	<0.001	-0.183 (0.029)	<0.001
BMI (vs. 18.5–24.9 kg/m^2)				
< 18.5	-0.001 (0.018)	0.971	0.001 (0.018)	0.969
≥ 25	-0.107 (0.011)	<0.001	-0.108 (0.011)	<0.001
Activity daily living, < 5 units (vs. ≥ 5)	-0.125 (0.012)	<0.001	-0.119 (0.012)	<0.001
Remaining teeth, < 20 tooth (vs. ≥20)	-0.230 (0.010)	<0.001	-0.228 (0.010)	<0.001
Comorbidity, yes (vs. no)	-0.026 (0.013)	0.053	-0.026 (0.013)	0.051
Current smoking (vs. never)	-0.242 (0.014)	<0.001	-0.240 (0.014)	<0.001
Household income, < 2.00 million yen/year (vs. 2.00–3.99)	-0.187 (0.011)	<0.001	-0.185 (0.011)	<0.001
Years of schooling, < 9 years (vs. 10–12)	-0.144 (0.010)	<0.001	-0.143 (0.010)	<0.001
No car-use (vs. use)	-0.124 (0.013)	<0.001	-0.128 (0.013)	<0.001
Convenience store	-0.002 (0.002)	0.247	-0.003 (0.002)	0.123
Land slope	-0.006 (0.002)	0.001	-0.004 (0.002)	0.084
	Var RE (SE)	ICC (SE)	Var RE (SE)	ICC (SE)
	0.005 (0.001)	0.006 (0.001)	0.008 (0.001)	0.008 (0.001)
Meat/fish				
Poor access (vs. good access)	0.019 (0.015)	0.194	-0.034 (0.005)	<0.001
Age	0.005 (0.0004)	<0.001	0.005 (0.0004)	<0.001
Men (vs. women)	-0.086 (0.005)	<0.001	-0.088 (0.005)	<0.001
Living alone (vs. with others)	-0.017 (0.008)	0.029	-0.017 (0.008)	0.031
Never married or others (vs. married)	-0.063 (0.014)	<0.001	-0.062 (0.014)	<0.001
BMI (vs. 18.5–24.9 kg/m^2)				
< 18.5	0.005 (0.008)	0.578	0.005 (0.008)	0.540
≥ 25	-0.022 (0.005)	<0.001	-0.023 (0.005)	<0.001
Activity daily living, < 5 units (vs. ≥ 5)	-0.041 (0.005)	<0.001	-0.039 (0.005)	<0.001
Remaining teeth, < 20 tooth (vs. ≥ 20)	-0.087 (0.004)	<0.001	-0.087 (0.004)	<0.001
Comorbidity, yes (vs. no)	-0.010 (0.006)	0.084	0.011 (0.006)	0.082
Current smoking (vs. never)	-0.013 (0.007)	0.062	-0.012 (0.007)	0.070
Household income, < 2.00 million yen/year (vs. 2.00–3.99)	-0.078 (0.005)	<0.001	-0.078 (0.005)	<0.001
Years of schooling, < 9 years (vs. 10–12)	-0.086 (0.005)	<0.001	-0.086 (0.005)	<0.001
No car-use (vs. use)	-0.011 (0.006)	0.062	-0.012 (0.006)	0.047
Convenience store	-0.0002 (0.001)	0.887	-0.0003 (0.001)	0.805
Land slope	0.001 (0.001)	0.443	0.002 (0.001)	0.213
	Var RE (SE)	ICC (SE)	Var RE (SE)	ICC (SE)
	0.004 (0.0004)	0.016 (0.002)	0.004 (0.0004)	0.016 (0.002)

Associations were assessed using a multilevel Tobit model† among 60,576 residents in urban/suburban areas.

β, beta coefficients; SE, standard error; Var RE, random effect variance in 336 school districts in urban/suburban areas; ICC, intercorrelation between school districts.

†Var RE (SE) and ICC (SE) in the null model was 0.012 (0.002) and 0.011 (0.002) for vegetables/fruits and 0.006 (0.001) and 0.023 (0.002) for meat/fish, respectively

Additional file 1

Table S4 Rural area intake frequencies of vegetables/fruits and meat/fish according to objective and perceived access

	Objective access		Perceived access	
	β **(SE)**	***p*****-Value**	***β*** **(SE)**	***p*****Vvalue**
**Vegetables/fruits**				
Poor access (vs. good access)	0.052 (0.053)	0.323	-0.053 (0.016)	0.001
Age	0.017 (0.001)	<0.001	0.017 (0.001)	<0.001
Men (vs. women)	-0.303 (0.017)	<0.001	-0.305 (0.017)	<0.001
Living alone (vs. with others)	-0.008 (0.027)	0.771	-0.007 (0.027)	0.801
Never married or others (vs. married)	-0.191 (0.053)	<0.001	-0.190 (0.053)	<0.001
BMI (vs. 18.5–24.9 kg/m^2)				
< 18.5	-0.039 (0.030)	0.190	-0.039 (0.030)	0.191
≥ 25	-0.064 (0.018)	<0.001	-0.064 (0.018)	<0.001
Activity daily living, < 5 units (vs. ≥ 5)	-0.162 (0.018)	<0.001	-0.158 (0.018)	<0.001
Remaining teeth, < 20 tooth (vs. ≥20)	-0.164 (0.018)	<0.001	-0.163 (0.018)	<0.001
Comorbidity, yes (vs. no)	-0.028 (0.022)	0.196	-0.029 (0.022)	0.181
Current smoking (vs. never)	-0.247 (0.025)	<0.001	-0.248 (0.025)	<0.001
Household income, < 2.00 million yen/year (vs. 2.00–3.99)	-0.134 (0.019)	<0.001	-0.133 (0.019)	<0.001
Years of schooling, < 9 years (vs. 10–12)	-0.104 (0.017)	<0.001	-0.104 (0.017)	<0.001
No car-use (vs. use)	-0.151 (0.024)	<0.001	-0.150 (0.024)	<0.001
Convenience store	0.006 (0.012)	0.635	0.003 (0.012)	0.840
Land slope	0.004 (0.003)	0.150	0.005 (0.002)	0.029
	Var RE (SE)	ICC (SE)	Var RE (SE)	ICC (SE)
	0.016 (0.004)	0.015 (0.003)	0.017 (0.004)	0.016 (0.004)
**Meat/fish**				
Poor access (vs. good access)	0.035 (0.033)	0.288	-0.020 (0.007)	0.007
Age	0.005 (0.001)	<0.001	0.005 (0.001)	<0.001
Men (vs. women)	-0.047 (0.007)	<0.001	-0.047 (0.007)	<0.001
Living alone (vs. with others)	-0.045 (0.012)	<0.001	-0.045 (0.012)	0.031
Never married or others (vs. married)	-0.046 (0.024)	0.054	-0.046 (0.024)	0.056
BMI (vs. 18.5–24.9 kg/m^2)				
< 18.5	0.005 (0.013)	0.714	0.005 (0.013)	0.713
≥ 25	-0.011 (0.008)	0.165	-0.011 (0.008)	0.158
Activity daily living, < 5 units (vs. ≥ 5)	-0.042 (0.008)	<0.001	-0.041 (0.008)	<0.001
Remaining teeth, < 20 tooth (vs. ≥ 20)	-0.063 (0.008)	<0.001	-0.063 (0.008)	<0.001
Comorbidity, yes (vs. no)	0.0003 (0.010)	0.971	-0.00002 (0.010)	0.998
Current smoking (vs. never)	-0.017 (0.012)	0.146	-0.017 (0.012)	0.139
Household income, < 2.00 million yen/year (vs. 2.00–3.99)	-0.083 (0.008)	<0.001	-0.083 (0.008)	<0.001
Years of schooling, < 9 years (vs. 10–12)	-0.071 (0.008)	<0.001	-0.071 (0.008)	<0.001
No car-use (vs. use)	-0.044 (0.011)	<0.001	-0.044 (0.011)	<0.001
Convenience store	-0.012 (0.007)	0.110	-0.013 (0.008)	0.076
Land slope	0.001 (0.002)	0.648	0.002 (0.001)	0.291
	Var RE (SE)	ICC (SE)	Var RE (SE)	ICC (SE)
	0.007 (0.001)	0.029 (0.006)	0.007 (0.002)	0.030 (0.006)

Associations were assessed using a multilevel Tobit model† among 22,808 residents in rural areas.

β, beta coefficients; SE, standard error; Var RE, random effect variance in 78 school districts in rural areas; ICC, intercorrelation between school districts.

†Var RE (SE) and ICC (SE) in the null model was 0.026 (0.005) and 0.023 (0.004) for vegetables/fruits and 0.006 (0.001) and 0.025 (0.005) for meat/fish, respectively

Additional file 1

Table S5 Evaluation of the association of food access with the prevalence of underweight and overweight individuals compared to those with a normal weight

	Good	Poor			
	**Case, n (%)**	**Case, n (%)**	**OR (95%CI)**	**Var RE (SE)**	**ICC (SE)**
Objective access					
Underweight	3772 (9.4)	1987 (8.9)	0.88 (0.81, 0.97)	0.011 (0.005)	0.003 (0.002)
Overweight	11010 (23.3)	6572 (24.4)	1.05 (0.98, 1.14)	0.021 (0.004)	0.006 (0.001)
Perceived access					
Underweight	4203 (9.0)	1556 (9.9)	1.06 (0.99, 1.13)	0.013 (0.006)	0.004 (0.002)
Overweight	13186 (23.7)	4396 (23.7)	0.97 (0.93, 1.02)	0.021 (0.004)	0.006 (0.001)

OR = odds ratio; CI = confidence interval; Var RE = random effect variance in 426 school districts; SE = standard error; ICC = intercorrelation between the school districts; Underweight, normal weight, and overweight were defined by body mass index < 18.5, 18.5–24.9, and ≥ 25 kg/m^2, respectively; In the null model, the Var RE (SE) and ICC (SE) was 0.015 (0.006) and 0.005 (0.002) for underweight and 0.031 (0.005) and 0.009 (0.015) for overweight; The association was evaluated using a multilevel logistic regression model adjusted for age, sex, family structure, marital status, activity daily living, remaining teeth, comorbidity, smoking status, household income, years of schooling, car use, urban/suburban area, convenience stores, and land slope among 80,012 residents.

Additional file 1

Table S6 Evaluation of the association of intake frequencies of vegetables/fruits and meat/fish with body mass index

	Vegetables/fruits				Meat/fish			
	β (SE)	*p*-value	Var RE (SE)	ICC (SE)	β (SE)	*p*-value	Var RE (SE)	ICC (SE)
Body mass index	-0.006 (0.001)	<0.001	0.070 (0.004)	0.014 (0.002)	-0.004 (0.001)	<0.001	0.074 (0.003)	0.025 (0.002)

β =beta coefficients; SE = standard error; Var RE = random effect variance in 426 school districts; ICC = intercorrelation between school districts; In the null model, the Var RE (SE) and ICC (SE) was 0.069 (0.004) and 0.013 (0.001) for vegetables/fruits and 0.074 (0.003) and 0.025 (0.002) for overweight; The association was evaluated using a multilevel regression model adjusted for age and sex among 80,012 residents.
